# Supplementary figures and images for: New Insights into the Interaction between Graphene Oxide and Beta-Blockers
Source: Nanomaterials (Basel). 2019 Oct 9;9(10):1429. doi: 10.3390/nano9101429 (PMC6835990; doi:10.3390/nano9101429)

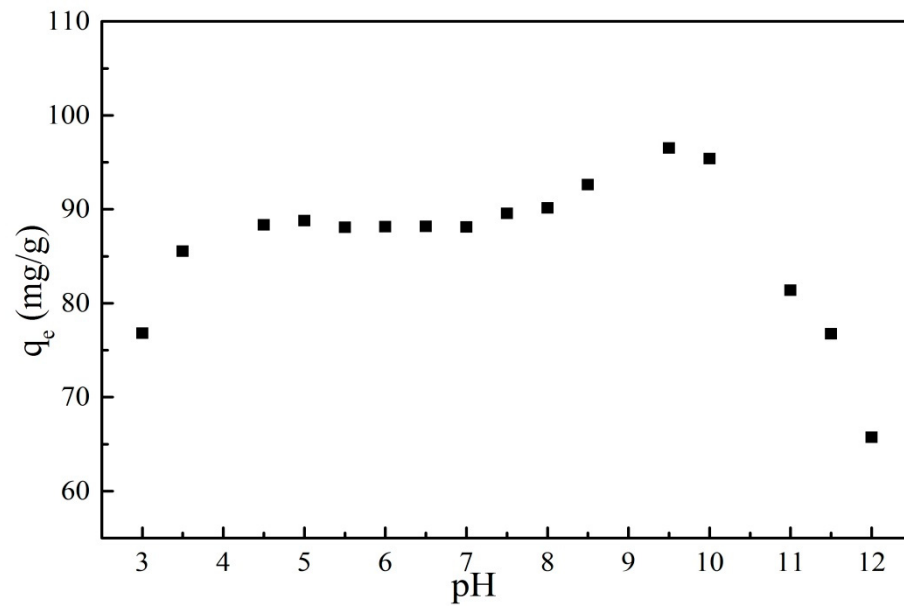

**Figure S1.** Effect of pH on the adsorption of GO for PRO ( $C_0=25$  mg/L,  $V=40$  mL,  $m=0.01$  g,  $t=24$  h).

Supplement: Supplementary file 1 [file nanomaterials-09-01429-s001.pdf]
